# Genome-Wide Differential Airway Gene Expression Analysis Identifies Genes Associated with COPD Comorbidities

**DOI:** 10.1007/s00408-025-00814-6

**Published:** 2025-04-26

**Authors:** Alen Faiz, Else A. M. D. ter Haar, Jorine E. Hartman, Corry-Anke Brandsma, Wim Timens, Janette K. Burgess, David F. Choy, Michele A. Grimbaldeston, Lowie E. G. W. Vanfleteren, Dirk-Jan Slebos, Maarten van den Berge, Simon D. Pouwels

**Affiliations:** 1 Respiratory Bioinformatics and Molecular Biology Group, University of Technology Sydney, Sydney, Australia; 2 Department of Pulmonary Diseases, University of Groningen, University Medical Center Groningen, Groningen, The Netherlands; 3 GRIAC Research Institute, University of Groningen, University Medical Center Groningen, Groningen, The Netherlands; 4 Department of Pathology and Medical Biology, University of Groningen, University Medical Center Groningen, Groningen, The Netherlands; 5 Genentech Inc., South San Francisco, CA USA; 6 COPD Center, Sahlgrenska University Hospital and Institute of Medicine, Gothenburg University, Gothenburg, Sweden

**Keywords:** COPD, Comorbidities, GWAS, Notch, Osteoporosis, Hypercholesterolemia

## Abstract

Chronic obstructive pulmonary disease (COPD) is often associated with the co-occurrence of extra-pulmonary diseases, yet the underlying pathophysiology of comorbidities is poorly understood. In COPD patients, the bronchial epithelium often displays cellular damage and is chronically inflamed. The current study aimed to identify differentially expressed genes in bronchial epithelium of COPD patients with and without comorbidities. To this end, a genome-wide differential gene expression analysis was performed on bronchial epithelial samples of 123 severe COPD patients with and without the following comorbidities: anxiety, atherosclerosis, depression, hypercholesterolemia, hypertension, muscle wasting, osteoporosis, and low BMI. COPD patients with osteoporosis displayed higher expression of *COL6A3* and lower expression of *PHEX*. Furthermore, COPD patients with hypercholesterolemia displayed a distinct bronchial epithelial gene expression profile, with 162 differentially expressed genes. No differentially expressed genes were identified for the other comorbidities. This study identified differentially expressed bronchial epithelial genes associated with osteoporosis and hypercholesterolemia in COPD patients.

## Introduction

Chronic obstructive pulmonary disease (COPD) is a severe and debilitating lung disease, characterized by chronic bronchitis, as well as emphysema. COPD is caused by a combination of chronic inhalation of toxic gases and particles, such as cigarette smoke and air pollution, and genetic susceptibility. Many COPD patients also develop extra-pulmonary conditions, such as cardiovascular diseases, metabolic diseases, and osteoporosis. A study by Vanfleteren et al*.* showed that over 97% of COPD patients developed comorbidities and half of all COPD patients developed 4 or more comorbidities [1]. The prevalence of a variety of comorbidities in COPD patients is higher than may be expected based on factors, such as age, social-economic status, body mass index, or smoking history alone, suggesting that those factors in combination with COPD provide a variety of biological mechanisms increasing the susceptibility of COPD patients to develop secondary diseases [2, 3]. Limited research has been performed aimed at identifying the biological mechanisms underlying the increased susceptibility of COPD patients to develop extra-pulmonary comorbidities. It has been described that pro-inflammatory factors, such as cytokines, chemokines, and damage-associated molecular patters (DAMPs), released from the damaged and inflamed lungs in COPD patients, spill over from the lungs into the circulation [4, 5]. Further, it has been suggested that these increased levels of circulating pro-inflammatory and cellular stress-inducing factors can contribute to the development of extra-pulmonary comorbidities, such as cardiovascular diseases or osteoporosis [4, 6]. Moreover, we recently showed that the somatic mutation frequency of specific genes is increased in COPD patients with cardiovascular comorbidities or osteoporosis [7]. The common etiology in smoke exposure and genetic predisposition may lead to specific associations for which findings in the bronchial epithelium can serve as biomarkers for risk of developing a specific type of comorbidity. Alternatively, differences in expression in the affected bronchial epithelium of COPD patients may either directly or indirectly affect the release of detrimental circulating factors, potentially contributing to the development of comorbidities. As a first step in unraveling the complex pathophysiology of COPD comorbidities, we performed a differential genome-wide gene expression analysis on bronchial epithelial samples of 123 severe COPD patients with or without one of the eight most prevalent comorbidities. The identified genes can provide targets for further studies investigating specific COPD comorbidities.

## Methods

We used available data from 123 ex-smoking stage 3–4 COPD patients included in the cross-sectional SHERLOCk (An integrative genomic approach to Solve tHe puzzle of sevERe earLy-Onset COPD, ClinicalTrails.gov: NCT04263961 and NCT04023409) study. None of the patients smoked for at least 2 months prior to sampling, nor did they have an exacerbation or lung infection 4 weeks prior to sampling.

Bronchial brush samples were available from all patients, which were used to isolate RNA for gene expression analyses. RNA-seq was used to obtain gene expression profiles as previously described [7, 8]. Comorbidities were assessed using a self-administered questionnaire and validated using the patients’ medical records. Exact definitions and inclusion criteria for each comorbidity have been described previously [9]. All participants provided written informed consent, and the study was approved by the Medical Ethical committee of the University Medical Center Groningen (UMCG), the Netherlands (METc 2016/572).

Genome-wide differential gene expression analyses were performed by comparing COPD patients with or without one of the eight most prevalent comorbidities, i.e., anxiety, atherosclerosis, depression, hypercholesterolemia, hypertension, muscle wasting, osteoporosis, and low body mass index (BMI < 21 kg/m^2^). Genes displaying a false discovery rate (FDR) lower than 0.05 were considered as having a statistically significant association. Local network cluster analysis was performed using the STRING database (http://string-db.org).

## Results

Out of the 123 severe COPD patients 71% (*n* = 87) were female. The mean age was 59.8 (± 7.1) years and the average smoking history was 39 (± 18.3) pack-years. Further, the mean FEV_1_ was 24.8% (± 6.5) percent predicted and the FEV_1_/FVC ratio was 0.28 (± 0.06).

In our cohort, 77% of the COPD patients had a comorbidity and 56% had multiple comorbidities. The average number of comorbidities in our cohort was two. Only comorbidities that were present in more than 10 patients were included in this study. The eight most prevalent comorbidities (*n* > 10) were as follows: anxiety (*n* = 19), atherosclerosis (*n* = 11), depression (*n* = 21), hypercholesterolemia (*n* = 11), hypertension (*n* = 23), muscle wasting (*n* = 30), osteoporosis (*n* = 22), and low BMI (*n* = 12).

In order to identify differentially expressed genes associated with extra-pulmonary COPD comorbidities, a genome-wide differential gene expression analysis was performed between severe COPD patients with and without a specific comorbidity. Genes displaying a genome-wide significant association were found for the comorbidities osteoporosis and hypercholesterolemia (Table 1). Two out of the 11 patients with hypercholesterolemia also had osteoporosis and were thus included in both analyses. No genome-wide associations were identified for the other six comorbidities.

**Table 1:** Genes associated with the COPD comorbidities, Osteoporosis and Hypercholesterolemia

	FC	p value	FDR	Gene name
**Osteoporosis**				
*COL6A3*	0.863	6.71E-07	0.012	Collagen type VI alpha 3 chain
*PHEX*	− 0.47	8.71E-06	0.076	Phosphate-regulating endopeptidase X-linked
**Hypercholesterolemia**				
*IGHG1*	− 3.517	3.15E-05	0.027	Immunoglobulin heavy constant gamma 1
*WIF1*	2.871	1.6E-05	0.027	WNT inhibitory factor 1
*GNG4*	− 1.833	2.36E-05	0.027	G protein subunit gamma 4
*TTN*	− 1.457	4.97E-05	0.032	Titin
*FHOD3*	1.323	2.99E-06	0.016	Formin homology 2 domain containing 3
*IGF1*	− 1.232	5.88E-05	0.032	Insulin-like growth factor 1
*VEGFC*	− 1.089	9.94E-05	0.041	Vascular endothelial growth factor C
*FJX1*	0.97	4.52E-06	0.016	Four-jointed box kinase 1
*GAS6*	0.937	5.15E-07	0.008	Growth arrest-specific 6
*LINC00475*	0.935	4.7E-05	0.032	Long intergenic non-protein coding RNA 475

For osteoporosis, the two top genes with the lowest p value are shown, for hypercholesterolemia from the 44 genome-wide significant genes, the top 10 genes with the highest effect size (Log2(FC)) are shown. FC is the Log2 (Fold change). P-value depicts the nominal p value and FDR represents the false discovery rate

**Fig. 1:**
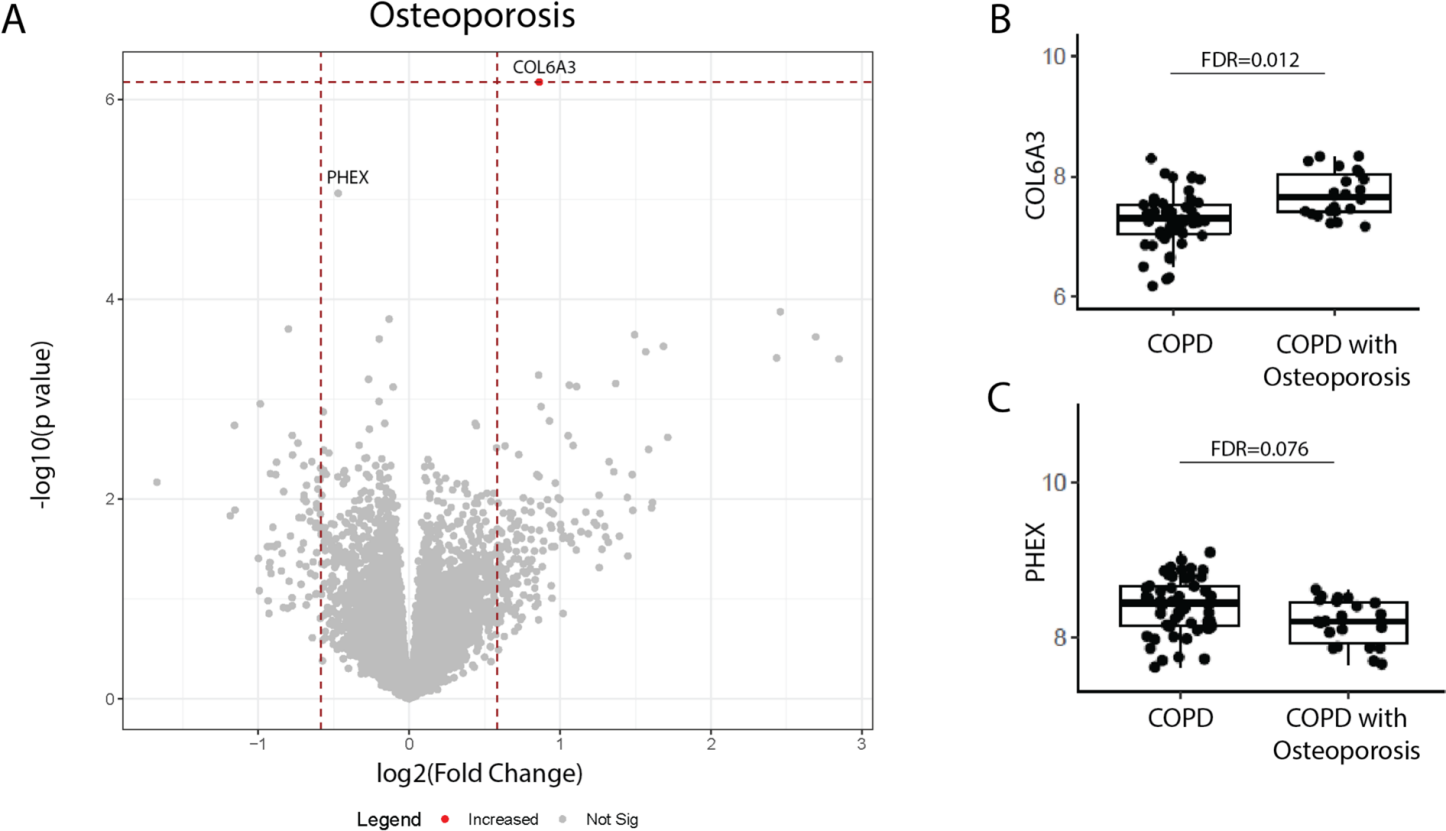
Genes associated with the COPD comorbidity osteoporosis. **A** Volcano plot showing genes of which the bronchial epithelial gene expression levels associated with COPD patients having osteoporosis as comorbidity. Significant (False Discovery Rate (FDR) < 0.05) genes are depicted in the figure as a red dot. The normalized bronchial epithelial gene expression of **B** *COL6A3* and **C** *PHEX* in COPD patients with (*n* = 22) and without (*n* = 52) osteoporosis

COPD patients with osteoporosis displayed higher bronchial epithelial gene expression of *COL6A3* (FDR = 0.012, nominal P-value = 6.7 × 10^–7^). Additionally, a trend was found for *PHEX* (FDR = 0.076, nominal P-value = 8.7 × 10^–6^), which was lower in COPD patients with osteoporosis (Fig. 1).

**Fig. 2:**
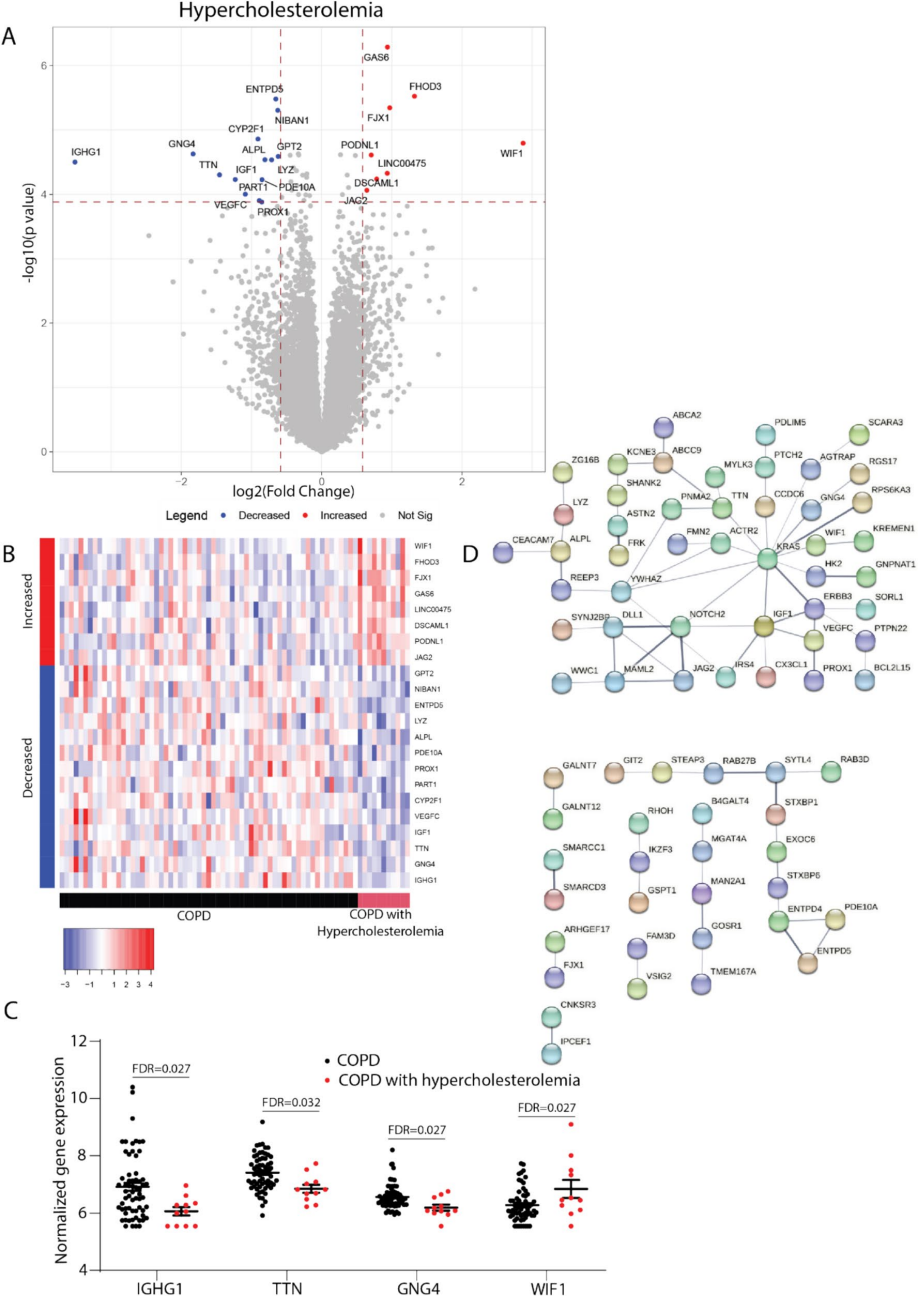
Genes associated with the COPD comorbidity hypercholesterolemia. **A** Volcano plot showing genes where the bronchial epithelial expression levels are associated with COPD patients having hypercholesterolemia as a comorbidity. Significant (False Discovery Rate (FDR) < 0.05) genes are depicted in the volcano plot. Red dots indicate genes with significantly higher gene expression, while blue dots indicate genes with significantly lower gene expression in COPD patients with hypercholesterolemia. **B** Heatmap showing genes with higher (red) and lower (blue) expression in COPD patients with hypercholesterolemia (red) compared to COPD patients without hypercholesterolemia (black). **C** The normalized bronchial epithelial gene expression of *IGHG1*, *TTN*, *GNG4*, and *WIF1* in COPD patients with (*n* = 11) and without (*n* = 63) hypercholesterolemia. **D** Local network cluster analysis showing the interactions between the 162 identified differentially expressed genes (FDR < 0.1) in COPD patients with and without hypercholesterolemia

Forty-four genes were identified that were significantly associated with having hypercholesterolemia as COPD comorbidity (FDR < 0.05). Additionally, 118 genes differentially expressed between COPD patients with and without hypercholesterolemia were identified with a FDR smaller than 0.1 (Fig. 2A). The bronchial epithelial gene expression profile of COPD patients with hypercholesterolemia displayed a distinct profile with both up- and down-regulated genes (Fig. 2B). The largest effect on gene expression was observed for *IGHG1* which was lower in COPD patients with hypercholesterolemia (Fig. 2C). Additionally, higher expression was observed in these patients for *WIF1* and lower expression of *GNG4* and *TTN*. Upon performing a local network cluster analysis (String analysis) on all 162 differentially expressed genes, an association was found with “Canonical and non-canonical Notch signaling” (*p* = 0.031) (Fig. 2D).

## Discussion

The current study identified genes that are differentially expressed in the bronchial epithelium of COPD patients with osteoporosis or hypercholesterolemia. This is the first step in identifying the role of the epithelium in the prediction of extra-pulmonary COPD comorbidities.

COPD patients with osteoporosis display a higher expression of *COL6A3* and a lower bronchial epithelial expression of *PHEX*. Collagen Type VI Alpha 3 Chain (COL6A3), one of three alpha-chains that together form the Collagen VI monomer, is an important extracellular matrix protein [10–12]. In COPD patients, an increased protein expression was found for Collagen VI in the submucosa and airways [13]. Additionally, an increase in the circulating levels of collagen VI fragments related to the production and degradation of Collagen VI, suggests an increased Collagen VI turn-over in COPD patients compared to healthy controls [14]. Interestingly, it was recently shown that COL6A3 can affect inflammation and apoptosis and enhance the osteogenic differentiation potential of bone marrow mesenchymal stem cells [15]. Therefore, it is tempting to speculate that the increased expression of COL6A3 in the lungs of COPD patients generates more circulating collagen VI fragments similar to what is seen in the development of osteoporosis. However, further studies aimed at identifying and correlating the circulating levels of COL6A3 in COPD patients with and without osteoporosis need to be performed. Additionally, we found that expression of Phosphate Regulating Endopeptidase X-Linked (PHEX), an important regulator of bone mineralization, was lower in COPD patients with osteoporosis. It has been shown that deleterious PHEX mutations can cause bone deformities, osteomalacia and fractures [16]. Future studies should further investigate the relationship between bronchial COL6A3 and PHEX expression and the development of COPD comorbidities.

The second COPD comorbidity for which differentially expressed genes were identified is hypercholesterolemia, which is defined as having high circulating levels of low-density lipoprotein (LDL) contributing to the development of atherosclerosis. In the current study, we identified that COPD patients with hypercholesterolemia have a distinct bronchial epithelial expression profile, with 162 genes being differentially expressed. Notch signaling pathway was identified upon analysis of the relationship between differentially expressed genes. Notch signaling is a fundamental signaling system that has many different functions in relation to homeostasis, tissue repair responses, proliferation and apoptosis. In the lungs Notch signaling is important for epithelial-to-mesenchymal transition (EMT), a process that can contribute to the development of lung fibrosis and COPD [16]. Additionally, several studies have found that Notch signaling can also contribute to goblet cell metaplasia and increased apoptosis in the lungs of COPD patients [17–19]. Future studies should be aimed at unraveling the exact role of the identified differentially expressed genes in COPD patients with hypercholesterolemia.

This study provides the first clues of the relevance of bronchial epithelial gene expression for association with osteoporosis and hypercholesterolemia in COPD patients. It should be noted that the identified associations do not need to be causally involved in the development of comorbidities. It is possible that the identified genes are associated with a common cause or a secondary disease-related factor. In case of common cause, it would be of interest to study whether the bronchial epithelial findings might have predictive value. Therefore, the results need to be validated in a second cohort. Future studies should elaborate on the function of the identified genes in relation to osteoporosis and hypercholesterolemia in COPD patients. Additionally, studies should be performed assessing whether the identified differences in gene expression are reflected in circulation or whether the identified genes affect other targets that can be identified in circulation or target organs. Moreover, it would be of interest to use single-cell sequencing to investigate whether comorbidities affect lung epithelial cell populations. In conclusion, this is the first study identifying differentially expressed genes in bronchial epithelium of COPD patients with and without osteoporosis or hypercholesterolemia. The identified genes associated with COPD patients with osteoporosis seem to be related to extracellular matrix turn-over and bone mineralization, while the genes associated with COPD patients with hypercholesterolemia are related to Notch signaling. The susceptibility genes and pathways identified in this study may provide valuable targets for future studies aimed at identifying the underlying pathophysiology of osteoporosis and hypercholesterolemia in COPD patients.

## Data Availability

All data are provided within the manuscript. Raw data can be obtained upon reasonable request to the corresponding author.
